# Detection of Interactions between Proteins by Using Legendre Moments Descriptor to Extract Discriminatory Information Embedded in PSSM

**DOI:** 10.3390/molecules22081366

**Published:** 2017-08-18

**Authors:** Yan-Bin Wang, Zhu-Hong You, Li-Ping Li, Yu-An Huang, Hai-Cheng Yi

**Affiliations:** 1 Xinjiang Technical Institutes of Physics and Chemistry, Chinese Academy of Science, Urumqi 830011, China; wangyanbin15@mails.ucas.ac.cn (Y.-B.W.); haichengyi@gmail.com (H.-C.Y.); 2 University of Chinese Academy of Sciences, Beijing 100049, China; 3 Department of Computing, Hong Kong Polytechnic University, Hong Kong, China; yahuang1991@gmail.com

**Keywords:** protein-protein interactions, Legendre moments, position specific scoring matrix, probabilistic classification vector machine

## Abstract

Protein-protein interactions (PPIs) play a very large part in most cellular processes. Although a great deal of research has been devoted to detecting PPIs through high-throughput technologies, these methods are clearly expensive and cumbersome. Compared with the traditional experimental methods, computational methods have attracted much attention because of their good performance in detecting PPIs. In our work, a novel computational method named as PCVM-LM is proposed which combines the probabilistic classification vector machine (PCVM) model and Legendre moments (LMs) to predict PPIs from amino acid sequences. The improvement mainly comes from using the LMs to extract discriminatory information embedded in the position-specific scoring matrix (PSSM) combined with the PCVM classifier to implement prediction. The proposed method was evaluated on *Yeast* and *Helicobacter pylori* datasets with five-fold cross-validation experiments. The experimental results show that the proposed method achieves high average accuracies of 96.37% and 93.48%, respectively, which are much better than other well-known methods. To further evaluate the proposed method, we also compared the proposed method with the state-of-the-art support vector machine (SVM) classifier and other existing methods on the same datasets. The comparison results clearly show that our method is better than the SVM-based method and other existing methods. The promising experimental results show the reliability and effectiveness of the proposed method, which can be a useful decision support tool for protein research.

## 1. Introduction

Proteins are a necessary component of the organism and are involved in almost all cellular activity in the organism. Protein-protein interactions (PPIs) play a very large part in most cellular processes. Studying protein interactions can provide insights into more biological processes, thereby better understanding the mechanisms of disease and developing disease-specific drugs. Hence, detection of the interactions of proteins has gradually become more important. In recent years, many high-throughput technologies have been designed for predicting PPIs, such as protein chips [1], immunoprecipitation [2,3], and yeast two-hybrid screening methods [4,5]. However, these methods are costly and time consuming. In addition, the above methods usually have high false positive rates and false negative prediction rates when dealing with large-scale experiments. Therefore, the development of reliable computational methods has important practical significance in promoting protein-protein interaction identification.

A number of computational methods have been proposed to detect PPIs based on different data types, such as phylogenetic profiles [6,7], literature mining knowledge [8], gene neighborhood [9,10], gene fusion [11,12], and sequence conservation [13,14]. However, these methods cannot work if the prior knowledge of a proteins is not available. Thanks to the rapid growth of protein sequence data, advances in computational methods for detecting PPIs have been promoted. Thus, many protein sequence-based approach have been proposed to identify PPIs. For example, Bock et al. [15] proposed a method that combined an SVM classifier with several physiochemical and structural descriptors to predict PPIs. Shen et al. developed a method based on SVM classifier and a conjoint triad feature extraction method. Chou et al. [16] introduced an approach named GO-PseAA to predicted PPIs that combined the method of pseudo-amino acid composition with the gene ontology. You et al. [17] present here a hierarchical model called PCA-EELM to predict PPIs only using the protein sequences. Several other methods have been reported in previous works, but there is still room for improving the accuracy and efficiency of PPI prediction [17,18,19,20,21,22,23,24,25,26,27,28,29,30].

In this paper, a novel sequence-based computational method was proposed for identifying PPIs that combines the probabilistic classification vector machine (PCVM) model with a novel protein sequence feature extraction scheme. More specifically, each amino acid sequence was represented as a position specific scoring matrix (PSSM) corresponding to physicochemical properties. Then, the Legendre moments (LMs) descriptor is applied to extract features from the PSSM that contain useful descriptive information. In addition, the principal component analysis (PCA) was used to reduce the influence of noise and feature dimensions. Finally, the probabilistic classification vector machine model was employed to detect PPIs. To assess the feasibility and effectiveness of the PCVM method, our proposed approach was implemented on two datasets, *Yeast* and *Helicobacter pylori*. The results show that the proposed method achieves satisfactory average accuracies of 96.37%, 93.48% respectively. To further validate the capabilities of our proposed approach, cross-species experiments were conducted on four separate datasets *Mix_Celeg*, *Mix_Ecoli*, *Mix_Hsapi*, and *Mix_Mmusc*. We also obtained good prediction accuracy in the cross-species experiments. In order to comprehensively evaluate the performance of the proposed method, we evaluated the PCVM classifier by comparing with the state-of-the-art support vector machine (SVM) classifier and other existing methods on the same dataset. The comparison results show that our method outperforms SVM and other previous methods.

## 2. Materials and Methodology

### 2.1. Godden Standard Datasets

We validate the proposed method on yeast and human datasets, which are collected from publicly-available interacting protein databases (DIP) [31]. In order to evaluate the proposed method accurately, we implemented a data preprocessing program to remove protein pairs with too high homologies and too few residues. As a result, we selected 5594 positive protein pairs for constructing positive datasets and 5594 negative protein pairs for constructing negative datasets from the *Yeast* dataset. Similarly, we selected 1458 protein pairs for constructing positive datasets and 1458 protein pairs for constructing negative datasets from the *H. pylori* dataset. Thus, the *Yeast* dataset contains a total of 11,188 protein pairs, and the *H. pylori* dataset contains a total of 2916 protein pairs.

### 2.2. Position-Specific Scoring Matrix

The position specific scoring matrix (PSSM) was first applied to detect distantly-related proteins based on sequences proteins. A PSSM of a query protein is a R × 20 matrix D = {$d_{ij}$: *i* = 1 ⋯ R *and j* = 1 ⋯ 20}, where the R represents the length of a protein sequence and the number 20 means 20 amino acids. As a scoring matrix, the element of PSSM represents a score of the $j_{th}$ amino acid in the $i_{th}$ position for the given query protein sequence, which was denoted as $d_{ij}=\sum_{k=1}^{20}p\left(u,k\right)\times w\left(v,k\right)$, where p(u,k) is the appearing frequency value of the $k_{th}$ amino acid at position u of the probe, and w(v,k) is the value of Dayhoff’s mutation matrix between $v_{th}$ and $k_{th}$ amino acids. Thus, a high score denotes a well-conserved position while, on the contrary, a low score represents a weakly-conserved position [32,33].

PSSM is widely used in a variety of biological tasks, such as prediction of disulfide connectivity, protein subcellular localization, protein quaternary structural attributes, and folding patterns. In this study, the PSSM is used for detecting PPIs. More specific, we employed the Position-Specific Iterated BLAST (PSI-BLAST) to translate each protein sequence into a PSSM. In order to obtain richer evolutionary information, the PSI-BLAST default value is set; in other words, the e-value parameter is set to 0.001 and iterated three times [34,35,36].

### 2.3. Legendre Moments

The moment in which the Legendre polynomial is used as the kernel function is defined as the Legendre moment, which is introduced by Teague. Legendre moments are a type of class orthogonal moment, which is widely used in image analysis and pattern recognition. They are used to achieve near-zero values of the redundancy measure in a set of moment functions. Thus, the moments become independent features of correspondence [37,38,39,40,41].

The 2-D Legendre moments of order (*m*, *n*), with image intensity function *f*(*x*; *y*), are defined as:

(1) $$L_{mn}=\mu_{mn}\int_{-1}^{1}\int_{-1}^{1}V_{m}\left(x\right)V_{n}\left(x\right)f\left(x,y\right)dxdy$$

where m,n=0,1,2,…, $\mu_{mn}=\left(2m+1\right)\left(2n+1\right)$/4, and the $m_{th}$ order LMs is given by:

(2) $$V_{m}\left(x\right)=\frac{1}{2^{m}m!}\frac{d^{m}}{dx^{m}}{\left(x^{2}-1\right)}^{m}=\frac{1}{2^{m}}\sum_{k=0}^{\left[m/2\right]}-1^{k}\left(\begin{array}{c} p\\ k \end{array}\right)\left(\begin{array}{c} 2\left(p-k\right)\\ p \end{array}\right)x^{p-2k}$$

which has the following orthogonality:

(3) $$\int_{-1}^{1}V_{m}\left(x\right)V_{n}\left(x\right)=\frac{2}{2m+1}\vartheta_{mn}$$

where $\vartheta_{mn}$ represents the Kronecher function.

An image of P×Q pixels with function f(i,j) can be expressed in discrete form as:

(4) $$L_{mn}=\mu_{mn}\sum_{i=1}^{P}\sum_{j=1}^{Q}h_{mn}\left(x,y\right)f\left(x,y\right)$$

where:

(5) $$h_{mn}\left(x,y\right)=\int_{x-\Delta x/2}^{x+\Delta x/2}\int_{y-\Delta x/2}^{y+\Delta x/2}V_{m}\left(x\right)V_{n}\left(x\right)dxdy$$

For the Legendre polynomials, there is an equation:

(6) $$\int V_{m}\left(x\right)dx=\frac{V_{m+1}\left(x\right)-V_{m-1}\left(x\right)}{2m+1}\;x\in \left[-1,1\right]$$

Hence, the following accuracy expression can be obtained by applying Equations (5) and (6):

(7) $$L_{mn}=\mu_{mn}\sum_{i=0}^{P-1}\sum_{j=0}^{Q-1}\frac{V_{m+1}\left(x+\frac{\Delta x}{2}\right)-V_{m-1}\left(x+\frac{\Delta x}{2}\right)-V_{m+1}\left(x-\frac{\Delta x}{2}\right)+V_{m-1}\left(x-\frac{\Delta x}{2}\right)}{2m+1}\times \frac{V_{n+1}\left(j+\Delta y/2\right)-V_{n-1}\left(j+\Delta y/2\right)-V_{n+1}\left(j-\Delta j/2\right)+V_{n-1}\left(j-\Delta j/2\right)}{2n+1}$$

**Figure 1:**
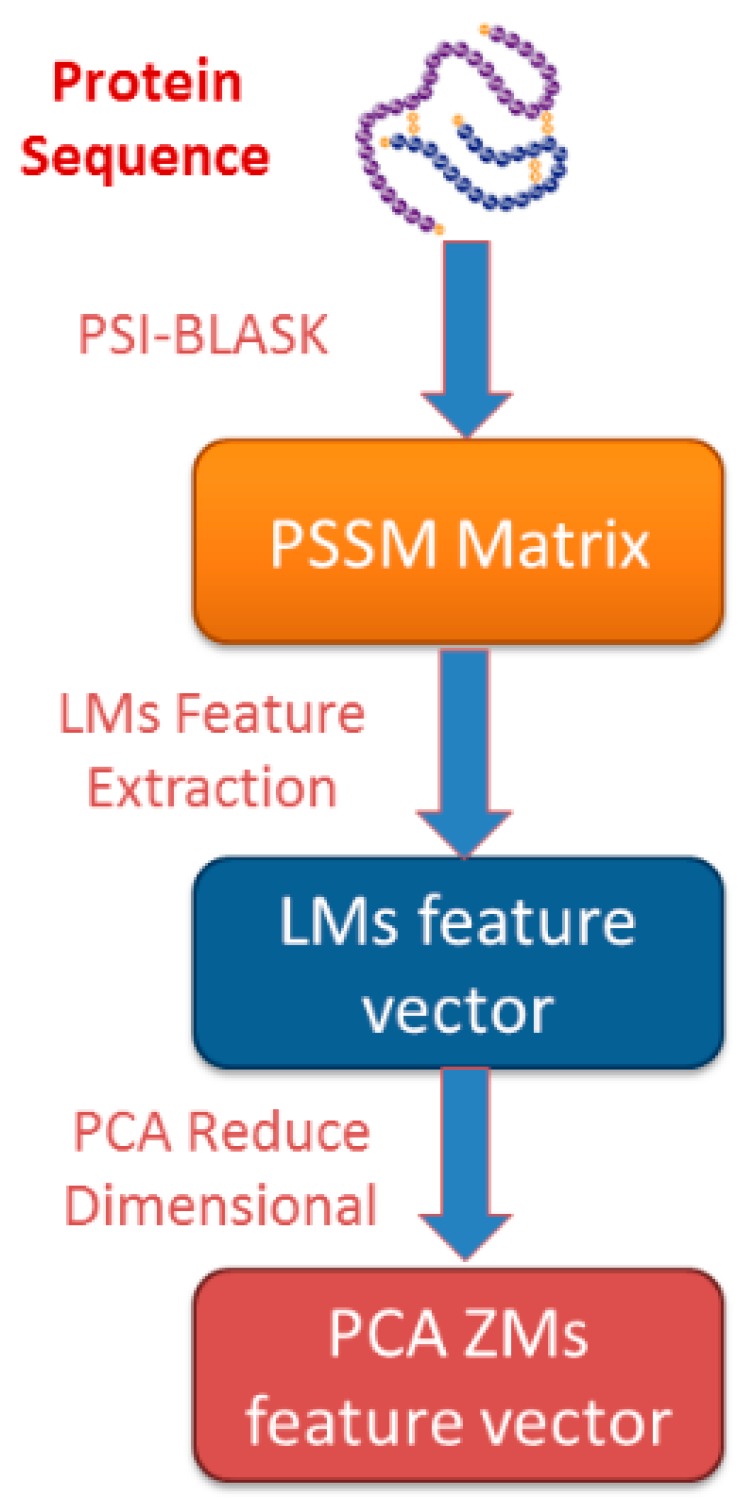
The flowchart of the proposed feature extraction method.

As a result, we obtained 441 features by using Legendre moments on PSSM of a given protein sequence. Therefore, each protein pair contains 882 features. The principal component analysis (PCA) method was employed to reduce the influence of noise and obtain distinguishing features. PCA transforms the feature into a new coordinate system, so that the maximum variance of the feature projection is located on the first coordinate. Finally, each protein pair was represented by only 100 features [42,43,44]. The flowchart of the proposed feature extraction scheme is displayed in Figure 1.

### 2.4. Related Machine Learning Models

When solving many pattern recognition problems, support vector machines (SVMs) are considered as a good alternative to traditional classifiers since, especially in the high-dimensional data space, it has better generalization ability. However, support vector machines have several obvious disadvantages: (1) the number of support vectors increases linearly with the size of the training set; (2) appropriate parameters are critical to support vector machine learning results and generalization ability. At present, parameter optimization is a bottleneck in the application of SVMs; and (3) support vector machines do not yield probabilistic outputs. These problems can be solved by another machine learning technique named relevance vector machines (RVM), which uses Bayesian inference to yield parsimonious solutions for probabilistic classification. Compared to the SVM, the Bayesian theory of the RVM avoids the parameter settings of the SVM that usually require cross-validation-based post-optimizations. However, a major problem with relevance vector machines is that they may lead to some untrustworthy vectors for system decision-making. Since the weights of positive and negative classes in RVM are determined by the zero mean Gauss distribution, this leads to certain training points that belong to the negative class being assigned positive weights, and vice versa. To overcome this problem, the PCVM classifier was proposed, which gives different prior weights to different classes of training points, i.e., in the positive samples, left-truncated Gaussian is used and, in the negative samples, right-truncated Gaussian is used. The PCVM provides many advantages relative to the above methods: (1) PCVM generates probabilistic results for each output; (2) PCVM is a sparse prediction model with less computational complexity, which leads to a more rapid performance in the testing stage; and (3) PCVM adopts an efficient parameter optimization procedure, based on probabilistic reasoning and an expectation maximization (EM) algorithm, which saves the cross-validation grid search effort and improves the performance.

### 2.5. PCVM Algorithm

Probabilistic classification vector machine (PCVM) is a sparse classification model, which aims to solve the problem of stability classification for relevance vector machines. In binary classification, the PCVM predictive model *f*(*x*; *w*) is generated by choosing a learning function f(·) which is determined by unknown parameters w that is determined by learning given a set of input-target training pairs ${\left\{x_{i},\;y_{i}\right\}}_{i=1}^{N}$, where $y_{i}$= {−1, +1}. The prediction *f*(*x*; *w*) consists of a linear combination of *M* basis functions:

(8) $$f\;\left(x;\;w\right)=\sum_{i=1}^{M}w_{i}\emptyset_{i,\theta }\left(x\right)+b$$

where the weight vector *W* = ${\left(w_{1},\ldots \ldots ,w_{M}\right)}^{T}$ is the parameter that decides the model, *b* represents the bias, and {$\emptyset_{1,\theta }\left(x\right),\ldots \ldots \emptyset_{M,\theta }\left(x\right)$} denotes the basis function, (wherein θ represents the parameter vector of the basis function).

The linear output is mapped to the binary output by using the probit link function Ω(x). The link function is the Gaussian cumulative distribution function and has the following form:

(9) $$\Omega \left(x\right)=\int_{-\infty }^{x}N\left(t|0,1\right)dt$$

After incorporating the kernel method with the probit link function, the model becomes:

(10) $$F\;\left(X;\;w,\;b\right)=\Omega \left(\sum_{i=1}^{M}w_{i}\emptyset_{i,\theta }\left(x\right)+b\right)=\Omega \left(\Phi_{\theta }\left(X\right)W+b\right)$$

A truncated Gaussian distribution as a prior is adopted over each weight $w_{i}$ as follows:

(11) $$p(W|\alpha )=\prod_{i=1}^{M}p\left(w_{i}|\alpha_{i}\right)=\prod_{i=1}^{M}N_{t}(w_{i}|0,\alpha_{i}^{-1})$$

A zero-mean Gaussian prior is used for *b*:

(12) $$p(b|\beta )=N(b|0,\;\beta^{-1})$$

The $N_{t}(w_{i}|0,\alpha_{i}^{-1})$ denotes a truncated Gaussian function, $\alpha_{i}$ is the precision of the corresponding parameter $w_{i}$, β represents the precision of the normal distribution of *b*. When $y_{i}=+1$, the truncated prior is a left-truncated Gaussian, and when $y_{i}=-1$, the prior is a right-truncated Gaussian. It can be denoted as:

(13) $$p(w_{i}|\alpha_{i})=\{\begin{array}{cc} 2N\left(w_{i}|0,\alpha_{i}^{-1}\right) & y_{i}w_{i}\geq 0\\ 0 & others \end{array}$$

We used the gamma distribution as the hyper prior of *α* and *β* and the employed EM algorithm for assigning the parameters of a PCVM model [45,46,47].

### 2.6. Initial Parameter Selection and Training

The PCVM has only one parameter, *θ*, which is automatically optimized in the training process. However, the EM algorithm converges easily to local maxima. The common method to avoid local extrema is to run the EM algorithm from different initialization points several times, and select the best initial point according to cross-validation error rate.

The best initial point of the PCVM is selected through the following procedure. The PCVM model was trained with nine initialization points over the first five training folds of each dataset. Hence, we obtain a 5 × 9 matrix of parameters that consist of these initial points, where the rows of the matrix denote the folds and the columns of the matrix represent the initializations. For each column, we reserve the initial point that produces the highest test accuracy, so that the matrix reduces from 45 to only five elements. Finally, we select the median over five parameters.

## 3. Results and Discussion

### 3.1. Performance Evaluation

For the purpose of measuring the performance of the proposed method, the following criteria (the set of metrics is valid only for the binary classification problem): the overall prediction accuracy (Acc), sensitivity (Sen), precision (Pre), and Matthews’s correlation coefficient (MCC) were calculated. They are defined as follows:

(14) $$\mathrm{Ac}=\frac{TP+TN}{TP+FP+TN+FN}$$

(15) $$\mathrm{Sn}=\frac{TP}{TP+TN}$$

(16) $$\mathrm{Pe}=\frac{TP}{FP+TP}$$

(17) $$\mathrm{Mcc}=\frac{\left(TP\times TN\right)-\left(FP\times FN\right)}{\sqrt{\left(TP+FN\right)\times \left(TN+FP\right)\times \left(TP+FP\right)\times \left(TN+FN\right)}}$$

where TP denotes the number of true positive that samples, having PPIs, that are predicted correctly, FP represents the number of false positive samples, having PPIs, that are predicted to be interaction. TN represents the number of true negative samples, true non-interacting pairs, that are predicted correctly. FN represents the number of false negative samples, true noninteracting pairs, that are predicted to be non-interacting. In addition, the receiver operating characteristic (ROC) curve is created and the area under an ROC curve (AUC) also is computed to further assess the performance [48,49]. For a more intuitive and easier-to-understand formulation about Equations (14)–(17), see Equation (14) of [50] or Equation (11) of [51], where a clear explanation was given for Ac, Sn, Pe, and Mcc, which is much easier for most experimental scientists to understand, particularly with respect to Mcc.

### 3.2. Assessment of Prediction

To validate the capabilities of the proposed model, we apply it on two approved datasets, *Yeast* and *H. pylori* datasets. In order to avoid the over-fitting in the experiment, the five-fold cross-validation is employed for performance evaluation.

**Figure 2:**
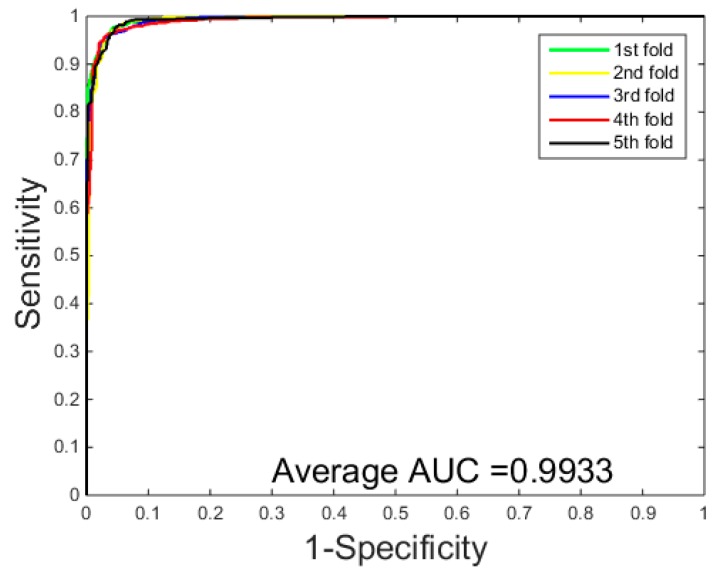
ROC curves performed of a probabilistic classification vector machines model (PCVM) on the *Yeast* dataset.

**Figure 3:**
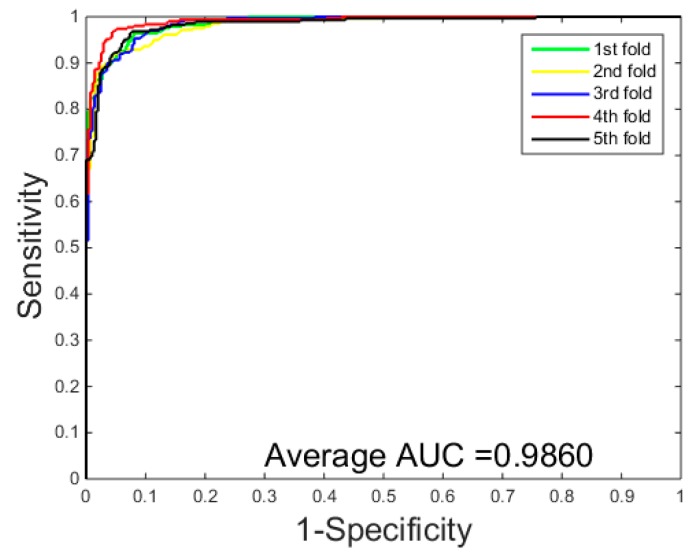
ROC curves performed of PCVM model on the *H. pylori* dataset.

When the proposed method was applied to detect PPIs on the *Yeast* dataset, it can be seen from Table 1 that we obtained the results of average accuracy, precision, sensitivity, and MCC of 96.37%, 96.60%, 96.15% and 93.00%, respectively. They standard deviations are 0.2%, 0.6%, 0.5% and 0.4%, respectively. When using the proposed method to predict PPIs on the *H. pylori* dataset, as can be seen from the Table 2, the proposed method also yielded good results of average accuracy, precision, sensitivity, and MCC of 93.48%, 94.40%, 95.46% and 87.79% and the standard deviations are 0.2%, 2.2%, 2.3% and 0.4%, respectively. The ROC curves performed on the two datasets are shown in Figure 2 and Figure 3. In order to further assess the performance of the PCVM method, the AUC values were calculated whose averages of the *Yeast* and *H. pylori* datasets are 99.33% and 98.60%, respectively.

The high prediction accuracy indicates that the PCVM classifier combining the LMs-PCA feature extraction strategy is effective and feasible for predicting PPIs. Furthermore, the low standard deviations suggest that the proposed method is robust and stable. The good performance is attributed to the feature extraction scheme, which not only preserves sufficient prior information, but also describes the sequence information of protein sequences. The PCVM classifier also has excellent predictive power.

### 3.3. Comparison the Proposed Method with the SVM-Based Approach

In order to see the feasibility of PCVM classifier, we compare it with the most advanced support vector machine (SVM) classifier. We employed LIBSVM tools [52] to implement the classification of SVM classifiers. For the sake of fairness, PCVM and SVM are executed on the same dataset using the same feature extraction scheme, respectively. The SVM parameters are c = 0.02 and g = 0.03 by using the grid search method, and other parameters use the default value.

**Figure 4:**
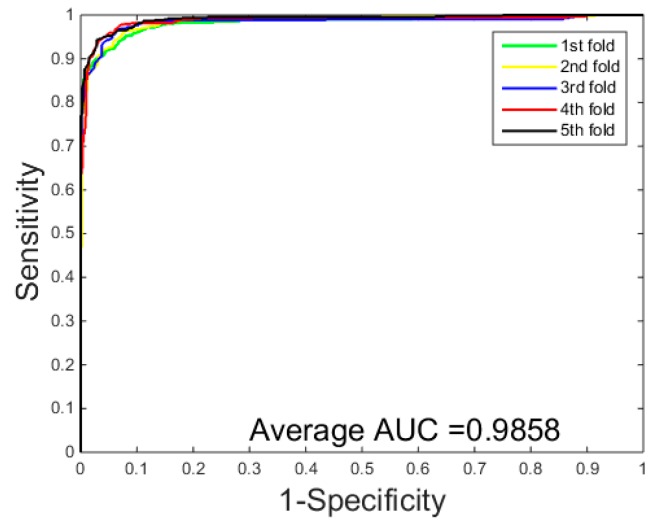
ROC curves performed of the support vector machine (SVM) on the *Yeast* dataset.

The prediction results of the SVM-based methods are listed in Table 3 and Table 4. They ROC curves of SVM method on the *Yeast* dataset are displayed in Figure 4. We can see from the Table 3 that the SVM technology achieved an average accuracy of 92.47%, an average sensitivity of 92.50%, an average precision of 93.01%, and an average Mcc of 86.00% on the *Yeast* dataset, while the prediction results of the PCVM technology achieved 96.37% average accuracy, 96.60% average sensitivity, 96.15% average precision, and 93.00% average Mcc on the *Yeast* dataset. Similar results were found in the Table 4, The prediction of *Helicobacter pylori* by the SVM method achieved an average accuracy of 90.50%, an average sensitivity of 92.74%, an average precision of 90.08%, and an average Mcc of 82.60%, while the prediction results by the PCVM classifier achieved an average accuracy of 93.48%, an average sensitivity of 94.40%, an average precision of 95.46%, and an average Mcc of 87.79%. The results of the comparison with SVM clearly illustrate that the PCVM classifier outperforms the SVM classifier in predicting protein interactions. In addition, from Figure 2 and Figure 4, the ROC curve of the PCVM method is significantly better than that of the SVM classifier. This shows that the PCVM classifier is reliable and accurate model and can be competent to predict PPIs in efficient manner. The following reasons lead to better prediction results of the PCVM classifier than the SVM classifier: (1) the computation of the PCVM kernel function is greatly reduced; (2) PCVM overcomes the deficiency of the kernel function satisfying the Mercer condition; and (3) PCVM provides more reliable support vectors. Due to these reasons, the PCVM method can yield higher predictions.

### 3.4. Performance on Independent Dataset

There is no doubt that our proposed approach has a strong PPIs predictive capability on *Yeast* and *H. pylori* datasets. However, for the performance of the proposed method, we have further verified by performing it on other four species, include *Mix_Celeg*, *Mix_Hsapi*, *Mix_Ecoli*, and *Mix_Mmusc*. In this experiment, the selected 11,188 samples of the *Yeast* data have been adopted to generate a prediction model, the samples from four other species are used to assess the performance of the prediction mode. Table 5 listed the experiment results. From the Table 5, we can find that our prediction model achieves a promising result of average prediction accuracy of 92.60%, 92.80%, 80.10% and 89.14%, respectively. These promising results not only demonstrate that the mechanism of yeast protein interaction may be similar to the other species, but also indicate that *Yeast* protein sequences can be used for protein interaction prediction on other species data. At the same time, it suggests that the proposed method has good generalizability.

### 3.5. Comparison with Other Methods

Many methods have been developed for the identification of PPI. In order to assess the performance of the model effectively, we also compared it with existing method. Results obtained by different methods on the *Yeast* dataset are listed in Table 6. As can be seen from Table 6, Guo’s work accuracy is as high as 89.33% and Zhou’s work performed well with a minimum standard deviation of 0.33%. In addition, Yang’s work achieved a higher accuracy, reaching 90.24%. It is worth mentioning that the proposed method yields the best performance in light of the sensitivity, precision, accuracy, and MCC at 96.37%, 96.60%, 96.15% and 93.00%, respectively. The standard deviations are 0.2%, 0.6%, 0.5% and 0.4%, respectively. The results demonstrate that our method has higher prediction accuracy [53,54,55,56,57,58,59,60].

Table 7 shows the results of several methods on *H. pylori* dataset. From the Table 7, it can be found that the proposed method achieves the highest performance with 93.48% prediction accuracy, which is 5.98% higher than the maximum value of the other five methods. The same is true for precision, sensitivity, and MCC. The improvement of prediction performance of our method may derive from the new feature extraction scheme, which can extract highly-discriminative information, and the PCVM classifier guarantees accurate and stable prediction [61,62,63,64]. 

## 4. Conclusions

Predicting the interactions between proteins is important for understanding the activity of complex cells from a molecular point of view. In this paper, a new computational method is reported for predicting protein-protein interactions using only protein amino acid information. The proposed prediction model is built by combining the PCVM classifier with the LMs-PCA descriptor. It can be seen from the experimental results that the prediction accuracy of this method is obviously higher than that of the previous methods. In addition, our proposed method has good prediction accuracy for cross-species independent datasets. The improvement of our approach is mainly attributed by the use of the probabilistic classification vector machine (PCVM) classifier and the Legendre moments-principal component analysis (LMs-PCA) descriptor from the position specific scoring matrix (PSSM). All these results show that our proposed approach is a very promising, reliable, and efficient support tool for future proteomic studies [65].

## Figures and Tables

**Table 1:** Five-fold cross-validation results using our proposed method on the *Yeast* dataset.

Testing Set	Acc (%)	Sn (%)	Pe (%)	Mcc (%)
1	96.67	97.52	95.93	93.55
2	96.33	96.59	95.93	92.93
3	96.25	96.40	96.24	92.78
4	96.42	95.78	96.93	93.09
5	96.17	96.69	95.74	92.63
Average	96.37 ± 0.2	96.60 ± 0.6	96.15 ± 0.5	93.00 ± 0.4

**Table 2:** Five-fold cross-validation results using our proposed method on the *H. pylori* dataset.

Testing Set	Acc (%)	Sn (%)	Pe (%)	Mcc (%)
1	93.48	94.74	92.15	87.81
2	93.48	89.72	96.56	87.75
3	93.14	89.97	96.42	87.20
4	93.65	89.94	97.88	88.09
5	93.66	92.61	94.27	88.12
Average	93.48 ± 0.2	94.40 ± 2.2	95.46 ± 2.3	87.79 ± 0.4

**Table 3:** Five-fold cross-validation results using the SVM-based method on the *Yeast* dataset.

Testing Set	Acc (%)	Sn (%)	Pe (%)	Mcc (%)
1	92.83	96.20	90.23	86.66
2	92.67	97.10	88.91	86.38
3	92.25	85.60	99.05	85.60
4	92.25	98.15	87.65	85.62
5	92.34	85.45	99.23	85.73
Average	92.47 ± 0.3	92.50 ± 6.4	93.01 ± 5.7	86.00 ± 0.5

**Table 4:** Five-fold cross-validation results using the SVM-based method on the *H. pylori* dataset.

Testing Set	Acc (%)	Sn (%)	Pe (%)	Mcc (%)
1	90.74	99.65	84.27	82.99
2	90.22	99.65	83.38	82.14
3	90.74	81.94	100.00	82.98
4	90.39	82.47	99.22	82.48
5	90.41	100.00	83.53	82.42
Average	90.50 ± 0.2	92.74 ± 9.6	90.08 ± 8.7	82.60 ± 0.4

**Table 5:** Prediction results of the proposed method on four other species.

Species	Test Pairs	Accuracy
*Mix_Celeg*	4013	92.60%
*Mix_Ecoli*	6954	92.80%
*Mix_Hsapi*	1412	80.10%
*Mix_Mmusc*	313	89.14%

**Table 6:** Practical predicting results of different methods on the *Yeast* dataset. N/A: Not Available.

Model	Testing Set	Acc (%)	Sen (%)	Pre (%)	MCC (%)
**Guo** [22]	ACC	89.33 ± 2.67	89.93 ± 3.68	88.87 ± 6.16	N/A
	AC	87.36 ± 1.38	87.30 ± 4.68	87.82 ± 4.33	N/A
**Yang** [23]	Cod1	75.08 ± 1.13	75.81 ± 1.20	74.75 ± 1.23	N/A
	Cod2	80.04 ± 1.06	76.77 ± 0.69	82.17 ± 1.35	N/A
	Cod3	80.41 ± 0.47	78.14 ± 0.90	81.66 ± 0.99	N/A
	Cod4	86.15 ± 1.17	81.03 ± 1.74	90.24 ± 1.34	N/A
**You** [17]	PCA-EELM	87.00 ± 0.29	86.15 ± 0.43	87.59 ± 0.32	77.36 ± 0.44
**Wong** [24]	RF-PR-LPQ	93.92 ± 0.36	91.10 ± 0.31	96.45 ± 0.45	88.56 ± 0.63
**Proposed Method**	PCVM	96.37 ± 0.20	96.60 ± 0.6	96.15 ± 0.5	93.00 ± 0.4

**Table 7:** Practical predicting results of different methods on the *H. pylori* dataset. N/A: Not Available.

Model	Acc (%)	Sen (%)	Pre (%)	MCC (%)
Nanni [25]	83.00	86.00	85.10	N/A
Nanni [26]	84.00	86.00	84.00	N/A
Nanni and Lumini [27]	86.60	86.70	85.00	N/A
Z-H You [17]	87.50	88.95	86.15	78.13
L Nanni [26]	84.00	84.00	84.00	N/A
Proposed Method	93.48	94.40	95.46	87.79
